# Furin Regulates the Alveolarization of Neonatal Lungs in a Mouse Model of Hyperoxic Lung Injury

**DOI:** 10.3390/biom13111656

**Published:** 2023-11-16

**Authors:** Shin Kato, Osuke Iwata, Hiroyuki Kato, Satoko Fukaya, Yukari Imai, Shinji Saitoh

**Affiliations:** 1 Department of Pediatrics and Neonatology, Nagoya City University Graduate School of Medical Sciences, 1 Kawasumi, Mizuho-Cho, Mizuho-Ku, Nagoya 467-8601, Japan; o.iwata@med.nagoya-cu.ac.jp (O.I.); fukaya6363@gmail.com (S.F.); ss11@med.nagoya-cu.ac.jp (S.S.); 2 Department of Experimental Pathology and Tumor Biology, Nagoya City University Graduate School of Medical Sciences, Nagoya 467-8601, Japan; h.kato@med.nagoya-cu.ac.jp; 3 Department of Pharmacy, Kinjo Gakuin University, Nagoya 463-8521, Japan

**Keywords:** proprotein convertase, alveologenesis, bronchopulmonary dysplasia

## Abstract

Despite advances in treatment options, such as corticosteroid administration and less invasive respiratory support, bronchopulmonary dysplasia (BPD) remains an important prognostic factor in preterm infants. We previously reported that furin regulates changes in lung smooth muscle cell phenotypes, suggesting that it plays a critical role in BPD pathogenesis. Therefore, in this study, we aimed to evaluate whether it regulates the alveolarization of immature lungs through activating alveolarization-driving proteins. We first examined furin expression levels, and its functions, using an established hyperoxia-induced BPD mouse model. Thereafter, we treated mice pups, as well as primary myofibroblast cell cultures, with furin inhibitors. Finally, we administered the hyperoxia-exposed mice pups with recombinant furin. Immunofluorescence revealed the co-expression of furin with alpha-smooth muscle actin. Hyperoxia exposure for 10 d decreased alveolar formation, as well as the expression of furin and its target, IGF-1R. Hexa-D-arginine administration also significantly inhibited alveolar formation. Another furin inhibitor, decanoyl-RVKR-chloromethylketone, accumulated pro-IGF-1R, and decreased IGF-1R phosphorylation in myofibroblast primary cultures. Finally, recombinant furin treatment significantly improved alveolarization in hyperoxia-exposed mice pups. Furin regulates alveolarization in immature lungs. Therefore, this study provides novel insights regarding the involvement of furin in BPD pathogenesis, and highlights a potential treatment target for ameliorating the impact of BPD.

## 1. Introduction

Despite improvements in survival opportunities following preterm births, approximately 40–50% of preterm infants born at <28 weeks of gestation develop bronchopulmonary dysplasia (BPD) [1], and 16% develop the most severe form of BPD [2,3]. Previous studies have shown that BPD is associated with serious morbidities, including home-oxygen therapy (adjusted odds ratio (OR), 3.4; 95% confidence interval (CI), 1.8–6.3) [4], as well as long-term neurodevelopmental impairments (OR, 2.81; 95% CI, 1.59–4.96) [5]. BPD prevention and treatment strategies include antenatal and postnatal corticosteroid administration, less-invasive surfactant administration, non-invasive positive pressure respiratory support, and caffeine administration [6]. Such revisions of respiratory care inevitably alter the pathological phenotypes of BPD, characterized by the arrest of secondary alveolar septa formation or “new BPD” [7,8]. 

This suggests that further refinements of treatments may need to address the disruption and arrest of alveolarization. We have previously reported that furin, a major proprotein convertase (PC), cleaves cyclic guanosine monophosphate-dependent protein kinase I (PKGI); the cleaved PKGI fragment modulates the smooth muscle phenotype, which plays a critical role in BPD pathogenesis [9]. 

PCs are serine proteases, and the family of PCs comprises nine members in humans. They cleave and activate precursor proteins, such as peptide hormones, growth factors, and enzymes [10]. They have been shown to play important roles in human homeostasis, as well as in a diverse array of pathophysiological states, including malignancy, inflammation, and infection [11,12,13]. Furin and PC5, which are members of the PC family that exist in the lungs and vascular smooth muscle cells, play important roles in cardiovascular diseases [14]. Furthermore, furin cleaves and activates the proproteins of Platelet derived growth factor-A, Insulin-like growth factor-1 (IGF-1), and Transforming growth factor-β, which are involved in alveolarization [15,16,17]. The identification of changes in its expression level and dysfunction in BPD pathogenesis may facilitate the establishment of strategies for BPD prevention, early diagnosis, and novel therapeutic options. Therefore, in this study, we tested the hypothesis that furin regulates the alveolarization of immature lungs through activating alveolarization-driving factors.

## 2. Materials and Methods

### 2.1. Antibody and Reagents

The anti-furin antibody was obtained from Invitrogen (Waltham, MA, USA; PA1-062), the anti-insulin-like growth factor I receptor (IGF-IR) antibody was obtained from Cell Signaling (Danvers, MA, USA; 9750), and the anti-phospho insulin-like growth factor I receptor (P-IGF-IR) antibody was obtained from Cell Signaling (Danvers, MA, USA; 3024). The anti α-smooth muscle actin antibody was obtained from Thermofisher (Waltham, MA, USA; 1A4; 53-9760-82). The Alexa Fluor 488 conjugated anti-alpha smooth muscle actin antibody was obtained from Invitrogen (Waltham, MA, USA; 53-9760-82), the Alexa Fluor 594 anti-rabbit antibody was obtained from Invitrogen (Waltham, MA, USA; A-11012), and the Alexa Fluor 488 Donkey anti- mouse IgG H&L antibody was obtained from abcam (Cambridge, UK; ab150109). Peroxidase-conjugated goat anti-rabbit and rabbit anti-mouse IgG antibodies were obtained from Sigma (St. Louis, MO, USA; A0545, A9044). To inhibit furin convertase in vivo and in vitro, hexa-D-arginine from Tocris (Bristol, UK; 4711), and the membrane-permeable furin inhibitor decanoyl-Arg-Val-Lys-Arg-chloromethyl ketone (dec-RVKRCMK; Cayman, Ann Arbor, MI, USA; 14965), were used. A recombinant furin was obtained from New England Biolabs (Ipswich, MA, USA; #P8077S).

### 2.2. Hyperoxia-Exposed Neonatal Mouse Lung Injury Model

The study protocol was reviewed and approved by the subcommittee for Research on Animal Studies at Nagoya City University (approval number H29M-20). Pregnant C57BL6J mice at E14 or E15 were purchased from Japan SLC (Hamamatsu, Japan), and housed at the animal center at Nagoya-City University. Within 12 h after birth, the mice pups and their mothers were randomly exposed to either air or 85% O_2_ in an acrylic chamber for 10 d. Oxygen concentration was monitored and controlled continuously with OXY-1-M (JIKCO, Nagoya, Japan). The mothers in the normoxic and hyperoxic chambers were exchanged every 48 h to decrease the effect of the hyperoxic environment on them. The animal experiments were performed in a controlled light cycle (7.5:16.5-h light-dark cycle) and temperature (23 ± 2 °C) environment. 

### 2.3. Tissue Collection and Preparation for Immunohistochemistry

After 10 d of hyperoxia exposure, the mouse pups were euthanized via the intraperitoneal injection of mixed anesthetic agents, medetomidine hydrochloride (Meiji Seika Pharma, Tokyo, Japan; 75 µg/mL), midazolam (SANDOZ, Tokyo, Japan; 0.4 mg/mL), and butorphanol (Meiji Seika Pharma, Tokyo, Japan; 0.5 mg/mL) [18]. Next, thoracotomy was performed to allow the lungs to collapse. Then, the right lung lobe was snap-frozen in liquid nitrogen for qRT-PCR and immunoblotting, while the remaining left lung lobe was cannulated via the trachea with a 0.61-mm outer diameter polyethylene tube (SP10, Natsume Seisakusho, Tokyo, Japan), and inflated with 4% paraformaldehyde supplemented with 0.1% glutaraldehyde in PBS at 25 cm H_2_O pressure for 30 min. Thereafter, the main bronchus was ligated while the lungs were distended. The pup was then submerged in the above-mentioned fixative for 3 d at 4 °C until examination. 

#### 2.3.1. Furin Inhibitor Administration in Neonatal Mouse

The normoxic mouse pups were subjected to intraperitoneal injections of the furin inhibitor hexa-D-arginine (D6R) at a dosage of 25 µg/pup, whereas the control group received phosphate-buffered saline (PBS) injections. The administration of these treatments commenced on postnatal day 2 (P2), and was repeated four times at intervals of every other day. At P10, the pups were euthanized for subsequent analysis.

#### 2.3.2. Recombinant Furin Administration in Hyperoxia-Exposed Mouse Pups

The mouse pups exposed to hyperoxia were intraperitoneally injected with recombinant furin (0.1 U/pup), while the control group received PBS. These treatments were commenced at P2, were repeated four times, and spaced at intervals of every other day. Subsequently, pups from both treatment groups were euthanized at P10 for further analysis.

### 2.4. Tissue Preparation and Immunohistochemistry

Fixed left lungs were embedded in paraffin, and sectioned using microtome at 3-μm thickness. Paraffin sections on glass slides were deparaffinized and stained with hematoxylin and eosin for histological and morphometric analyses. For immunohistochemistry, antigen retrieval was performed through heating the slides at 100 °C for 20 min in citric acid (pH 6.0). After blocking the lung sections with 1% bovine serum albumin, and incubation with 1:100 anti-furin antibody, the slides were stained via Leica Bond-Max automation using Leica Refine detection kits (Leica Biosystems, Wetzlar, Germany).

### 2.5. Morphometric Analysis

Alveolar maldevelopment was determined based on mean linear intercept (Lm) and tissue volume density (TVD) using light microscopy. Lm, defined as the mean length of line segments on random test lines spanning the airspace between the intersections of the line with the alveolar surface [19], was obtained through dividing the total length of the line drawn across the lung section by the total number of intercepts. TVD, which was expressed as the proportion of lung tissue (alveolar ducts and sacs) in the lungs as previously described [20], was evaluated using a 10 × 10 grid with 100 evenly spaced points, 25 nm apart. Lm and TVD were assessed in 3–5 non-overlapping lung parenchyma fields in one tissue section per animal and, for each condition, at least five animals were examined. 

### 2.6. Primary Myofibroblast Cell Culture

For immunocytochemistry and protein detection, primary myofibroblast cell cultures were prepared using P1 mouse pups [21]. After euthanizing the pups using the mixed anesthetic agents described above, the pulmonary vessels of the pups were repeatedly flushed with PBS containing 0.1 M sodium citrate. The trachea, major bronchi, and major vessels were then removed using a dissecting microscope. This was followed by the mincing of the peripheral lung parenchyma, and processing using a lung cell dissociation kit (130-095-927, Miltenyi, Auburn, CA, USA) at 37 °C for 30 min with agitation. The lung digests were centrifuged at 300× *g* for 10 min, and the cell pellets obtained were resuspended in complete low-glucose Dulbecco’s modified Eagle’s medium (DMEM) with 10% heat-inactivated fetal bovine serum (FBS), penicillin, and streptomycin. Next, the resuspended cells were then placed in 10 cm Petri dishes for 30 min to remove macrophages and adherent lymphoid cells. Unadhered cells were collected and cultured on glass bottom plates or six well plates. Epifluorescence microscopy was performed to confirm myofibroblast identity based on their typical morphology and the expression of α-smooth muscle actin (SMA), as determined via reactivity with an antibody (1A4; Thermofisher, Waltham, MA, USA; 53-9760-82). Subconfluent primary cultures of these cells were used for subsequent cell staining and protein detection.

### 2.7. Furin Detection in Myofibroblast

Primary myofibroblast cells, derived from P1 mouse pups, were seeded at a density of 0.1 × 10^6^ cells/cm^2^ in glass bottom plates. The seeding medium comprised complete medium of 10% FBS in low glucose DMEM supplemented with antibiotics. On the following day, the cells were washed gently with PBS, and then fixed with 4% formalin in PBS at 37 °C for 15 min, permeabilized with methanol for 10 min, and blocked with 1% goat serum in PBS containing 0.1% Tween 20 overnight. The next day, the cells were incubated overnight either anti-furin primary antibody or Alexa Fluor 488 conjugated anti-alpha smooth muscle actin. Subsequently, Alexa Fluor 594-conjugated anti-rabbit antibody was applied, with or without DNA-binding 4′-6-diamidino-2-phenylindole (DAPI) to identify the nuclei. Wide-field epifluorescence microscopy was employed for imaging the cells.

### 2.8. Furin Inhibitor Treatment on Primary Myofibroblast Cell Culture

Primary myofibroblast cells, derived from P1 mouse pups, were seeded at a density of 0.3 × 10^6^ cells/cm^2^ in 6-well plates in complete medium of 10% FBS in low glucose DMEM supplemented with antibiotics. The following day, the cells were washed briefly with culture medium, and then the cells were subjected to serum starvation through exposure to media containing 0.2% FBS in low glucose DMEM supplemented with 50 μM of dek-RVKRCMK for 24 h. After overnight incubation, the cells were treated once again with 10% FBS DMEM, with or without dek-RVKRCMK, for an additional 24 h. Subsequently, the cells were gently washed with PBS, and then harvested using radioimmunoprecipitation assay (RIPA) buffer (50 mM Tris-HCl, pH 7.4, 150 mM NaCl, 0.5% octylphenoxypolyethoxyethanol, 1 mM dithiothreitol, 0.5% sodium deoxycholate, and 0.1% sodium dodecyl sulfate (SDS), supplemented with a protease inhibitor cocktail (Sigma, St. Louis, MO, USA; P8340) and phosphatase inhibitor (Sigma; P5726). The cell lysates were then processed for immunoblotting. After determining the protein concentration in each sample with the bicinchoninic acid-based protein assay method (Pierce Biotechnology, Waltham, MA, USA; 23227), 20 μg of each sample was utilized for immunoblotting analyses.

### 2.9. qRT-PCR Analysis

Whole lung samples underwent pretreatment with RNA stabilizing solution (QIAGEN, Hilden, Germany; 76104), and were then subsequently snap-frozen for RNA extraction. The RNeasy Mini Kit (QIAGEN, 74104) was employed for extraction following the manufacturer’s instructions. For cDNA synthesis, 1 μg of total RNA was utilized, employing SuperScript IV First-Strand Synthesis System (Invitrogen, Carlsbad, CA, USA; 18091050). The resultant cDNA was then subjected to amplification using the FastStart Essential DNA Green Master Mix (Roche, Mannheim, Germany; 06402712001) on a Light Cycler 96 system (Roche). The primer pairs used for amplification were as follows: furin, forward 5′- CAGAAGCATGGCTTCCACAAC-3′, reverse 5′- TGTCACTGCTCTGTGCCAGAA-3′; β-actin, forward 5′- GTGACGTTGACATCCGTAAAGA-3′, reverse 5′- GCCGGACTCATCGTACTCC-3′. Each sample underwent triplicate analysis under the same conditions. The standard curve was obtained by making a stepwise dilution of the cDNA from the sample being measured. Relative quantification was performed using the PCR efficiency derived from the standard curve, and the data were normalized to internal control, the β-actin.

### 2.10. Protein Isolation and Immunoblotting

The frozen right lobe of the lungs was crushed under liquid nitrogen and dissolved in RIPA buffer with a protease inhibitor cocktail. The cultured cells were collected using RIPA buffer supplemented with protease inhibitor cocktail and a phosphatase inhibitor (Sigma; P5726). The protein concentration in each sample was then determined using the bicinchoninic acid-based protein assay method. Approximately 50 µg of proteins in each sample were subjected to 7.5% reducing SDS–polyacrylamide gel electrophoresis. Proteins were transferred onto polyvinylidene fluoride membranes (Millipore, Burlington, MA, USA; IPVH00010), and the membranes were blocked with 3% nonfat dry milk in Tris-buffered saline containing 0.1% Tween 20. After overnight incubation at 4 °C with primary antibody, immunodetection was performed using the enhanced chemiluminescence method (Amersham Biosciences, Amersham, UK; RPN2232). The analysis of normalized densitometric data was performed using Image J software version 1.52a (NIH, Bethesda, MD, USA), and expressed in arbitrary units.

### 2.11. Statistical Analysis

All statistical analyses were performed using SPSS software version 26.0 (SPSS, Chicago, IL, USA). Data were presented as mean ± standard deviation, and treatment groups were compared using the Mann–Whitney U test or Kruskal–Wallis test followed by the Mann–Whitney U test. Statistical significance was set at *p* < 0.05. 

## 3. Results

### 3.1. Furin Expression in Developing Mouse Lungs

**Figure 1:**
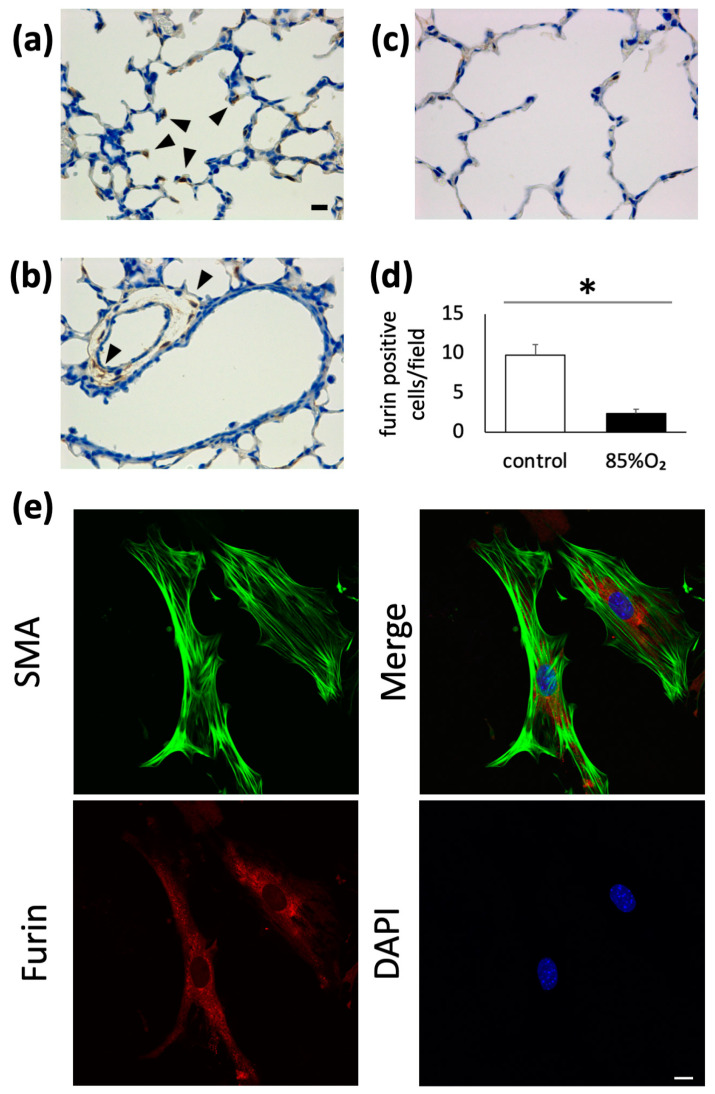
Furin expression in developing mouse lungs. Furin-positive cells (arrow head) were detected in P10 mice pups via immunohistochemistry using an anti-furin polyclonal antibody and observed under an optical microscope (**a**). Furin immunoreactivity was also detected in vascular smooth muscle cells (arrow head), but absent in airway epithelial cells (**b**). Furin expression in developing mouse lungs was downregulated following mice pup exposure to 85% O_2_ for 10 d; Scale bar, 10 μm (**c**). Number of furin-positive cells in air- or 85% O_2_-exposed mice pups was assessed (n = 5 animals in each group, * *p* < 0.05) (**d**). Furin was confirmed to be co-expressed with α-smooth muscle actin in cells isolated from the peripheral lungs of P1 mice; Scale bar, 20 μm (**e**).

To confirm furin expression in developing mouse lungs, we performed immunohistochemistry on P10 mouse pups using an anti-furin polyclonal antibody and observed under an optical microscope. As shown in Figure 1, furin-positive cells were observed at the tips of the secondary alveolar septa (Figure 1a). We observed furin immunoreactivity in vascular smooth muscle cells, but not in airway epithelium (Figure 1b). Our results also showed a decrease in the number of furin-positive cells in the lungs of mice that were exposed to 85% oxygen for 10 days (Figure 1c). Additionally, cell counts revealed that the number of furin-positive cells was significantly lower in the oxygen-exposed group (n = 5, *p* < 0.05) (Figure 1d). To further determine the specific cell types in which furin is expressed, we used immunofluorescence on primary cultures prepared from mouse peripheral lung cell suspension. Furin was confirmed to be co-expressed in SMA-positive cells (Figure 1e). 

### 3.2. Downregulated Furin Expression in Hyperoxia-Exposed Neonatal Mouse Lungs

**Figure 2:**
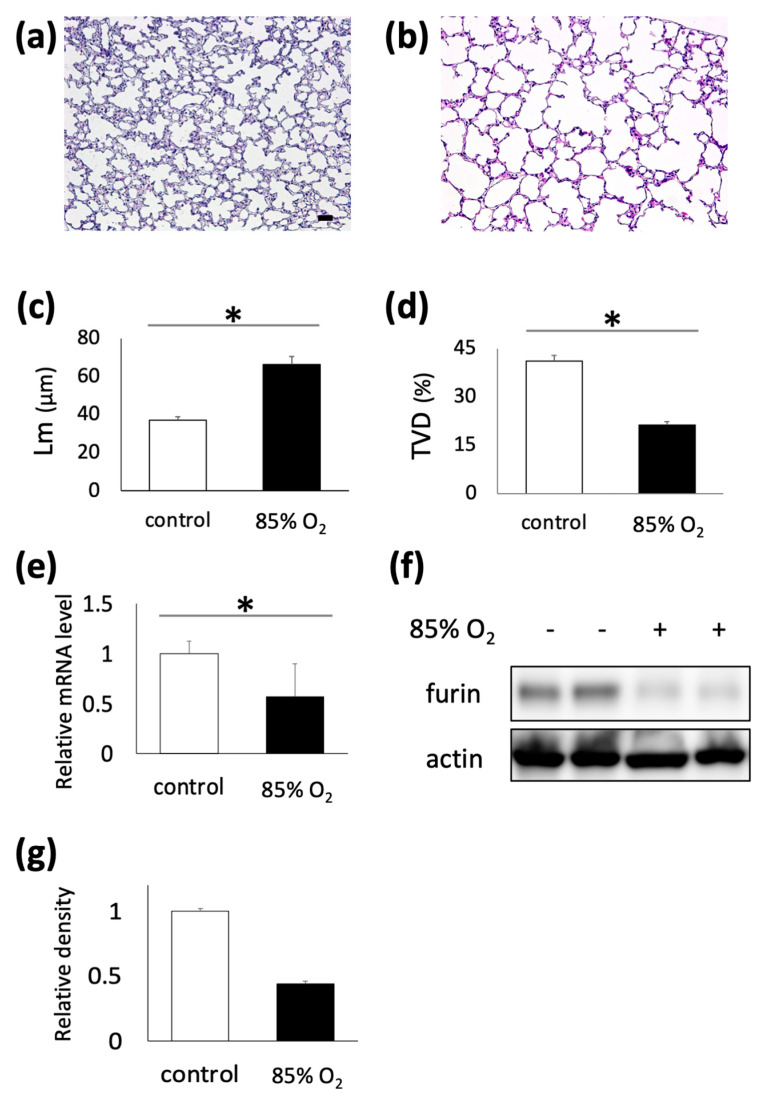
Downregulated furin expression in hyperoxia-exposed developing mouse lungs. C57BL6J mice pups and their mothers were exposed to 85% oxygen within 12 h of birth to P10. Hyperoxia exposure impaired alveolar development compared to the control on fixed sections of lungs in each treatment group, ((**a**); control, (**b**); hyperoxia), and the changes in hyperoxic mice pups were confirmed via Lm (**c**) and TVD (**d**) measurements (control, n = 7; hyperoxia, n = 5; * *p* < 0.05). qRT-PCR revealed a reduction in mRNA levels of furin in the lungs of mice exposed to hyperoxia (**e**). Immunoblotting of whole lung lysates from each group showed decreased furin immunoreactivity in hyperoxia-exposed mouse lungs (**f**) and densitometry results is shown (**g**); Scale bar, 50 μm (**a**). Lm, mean linear intercept; TVD, tissue volume density.

To confirm the influence of hyperoxia on neonatal mouse lungs and furin expression, we used a hyperoxia-induced mouse lung injury model. Hyperoxia exposure for 10 d significantly reduced alveoli formation compared to the control (Figure 2a,b), as confirmed by the increased Lm and decreased TVD in mouse pups exposed to hyperoxia (Figure 2c,d, control, n = 7; 85% O_2_, n = 6; *p* < 0.05). We also confirmed that mRNA expression level of furin determined using q RT-PCR showed significant decrease following hyperoxic exposure (Figure 2e). Furthermore, immunoblotting using whole lung lysate confirmed that furin expression was downregulated in the lungs of mice that were exposed to hyperoxia for 10 days (Figure 2f). The densitometry results obtained are shown in Figure 2g.

### 3.3. Downregulated IGF-1R Expression in Hyperoxia-Exposed Neonatal Mouse Lungs

**Figure 3:**
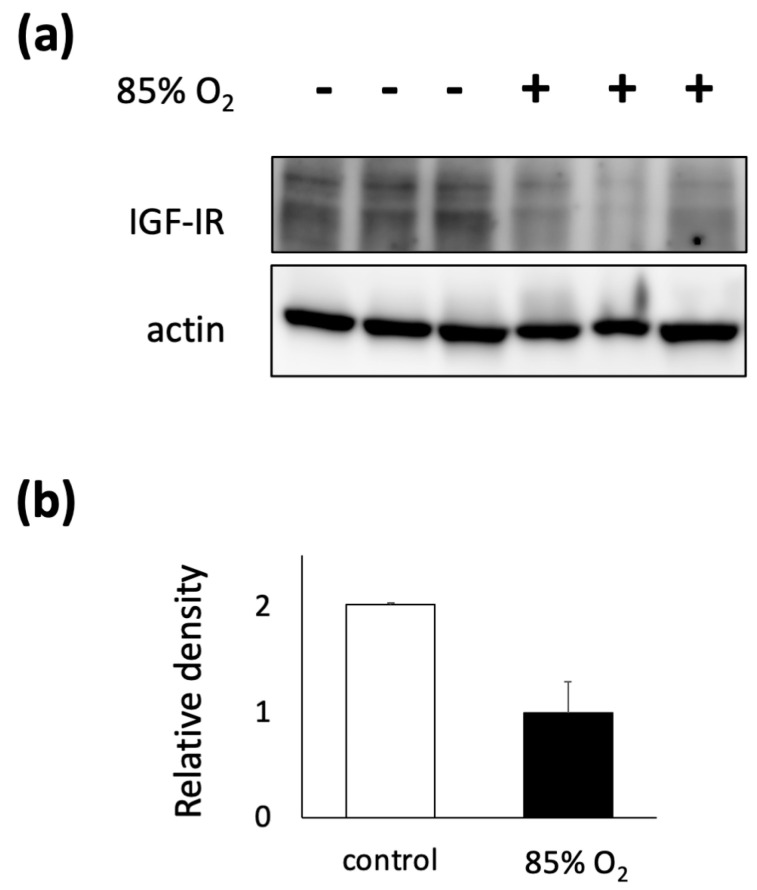
Downregulated expression of a furin target protein in hyperoxia-exposed mouse lungs. Immunoreactivity of IGF-1R, a protein cleaved and matured by furin, was confirmed using whole lung lysates from hyperoxia-exposed mouse lungs. IGF-1R expression was downregulated in hyperoxia-exposed mouse lungs compared to the control on P10 (**a**), and densitometry results is shown (**b**).

To further confirm the changes in the expression levels of growth factors associated with alveolar septal formation among furin target proteins, IGF-1R expression was detected via immunoblotting. IGF-1R was selected because it is a membrane-bound protein, whereas other growth factors targeted by furin are secretory proteins. Immunoblotting using whole lung lysates from hyperoxia-exposed mouse lungs showed a decrease in the expression level of IGF-1R immunoreactivity (Figure 3a). The densitometry results obtained are shown in Figure 3b.

### 3.4. D6R Reduced Alveoli Formation in Developing Mouse Lungs

**Figure 4:**
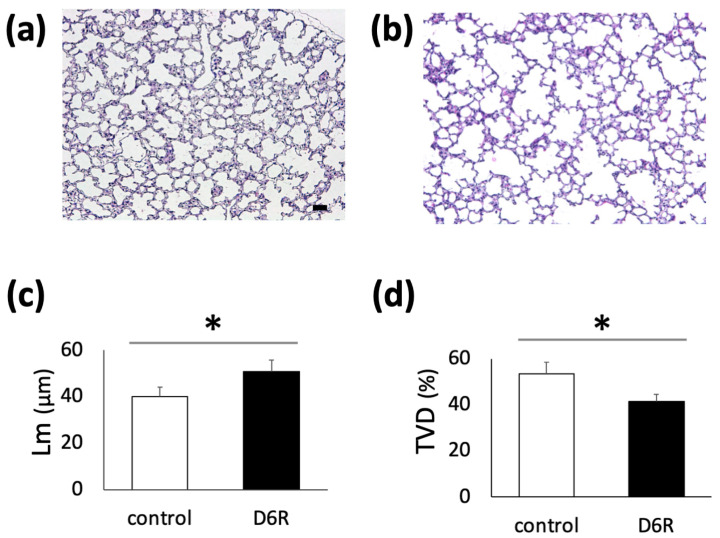
Impaired alveolar development using a furin inhibitor, hexa-D-arginine (D6R), in developing mouse lungs. Mice pups were intraperitoneally injected a peptide furin inhibitor, D6R, four times every 2 d from P2, and the pups were euthanized at P10. Lung morphology was examined ((**a**); control, (**b**); D6R-treated), and changes in D6R-treated mouse lungs were confirmed via Lm (**c**) and TVD (**d**) measurements; Scale bar, 50 μm (control, n = 7; D6R, n = 6; * *p* < 0.05). Lm, mean linear intercept; TVD, tissue volume density.

To determine whether furin plays an important role in alveolar septa formation, a peptide furin inhibitor D6R was injected intraperitoneally every 2 d from P2, and the mice were euthanized at P10. The D6R-treated group showed decreased alveoli formation compared to the control group at P10 (Figure 4a,b). Additionally, the degree of alveolar formation was confirmed using Lm and TVD measurements (Figure 4c,d; control, n = 6; D6R, n = 7; * *p* < 0.05).

### 3.5. Furin Inhibitor, Decanoyl-RVKR-Chloromethylketone (Dec-CMK), Inhibited the Activation of Pro-IGF-1R and Reduced Its Signal

**Figure 5:**
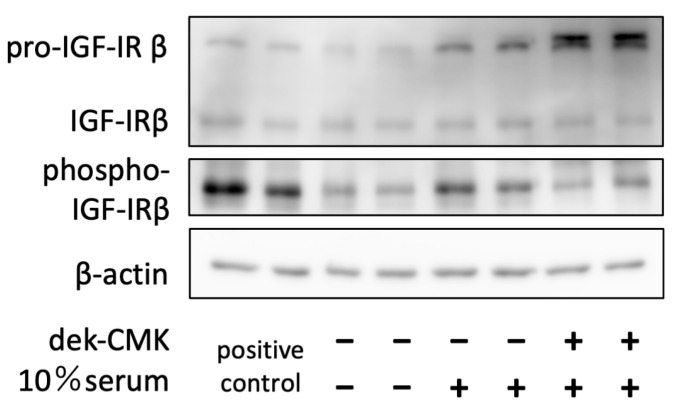
Furin inhibition accumulates IGF-1R proprotein and decreases its signaling. Subconfluent primary myofibroblast cultures of P1 mice pup lungs were serum-starved for 24 h under treatment with a furin inhibitor, decanoyl-Arg-Val-Lys-Arg-chloromethylketone (dek-RVKR-CMK), and then allowed to recover for another 24 h with continued inhibitor treatment. After cell harvesting, the immunoreactivity of the pro-IGF-1R and phospho-IGF-1R was determined in cell lysates using specific antibodies and immunoblotting.

To further explore the effect of furin inhibition on the activation and signaling of its target protein, we treated myofibroblast primary culture cells with dec-CMK, examined pro-, activated-IGF-1R expression, as well as phosphorylation. The immunoblotting of cell lysates with/without dec-CMK treatment showed the accumulation of pro-IGF-1R immunoreactivity (Figure 5). The double bands of pro-IGF-1R immunoreactivity indicate the presence of the endoplasmic reticulum- and Golgi apparatus-form, which are produced during intracellular protein transport and post-translational modification in each compartment. Furthermore, IGF-1R phosphorylation was also inhibited in dec-CMK-treated cells.

### 3.6. Treatment with Recombinant Furin Rescued Deteriorated Alveologenesis in Hyperoxia-Exposed Neonatal Mice

**Figure 6:**
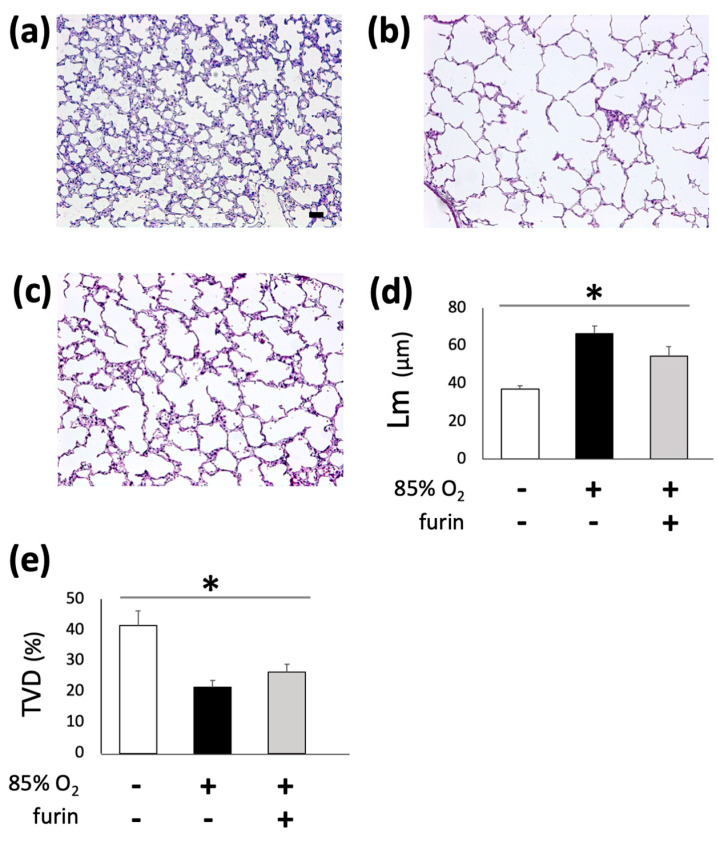
Recombinant furin treatment is associated with improved alveolar development in developing mouse lungs exposed to hyperoxia. Exposure to 85% O_2_ for 10 d was associated with decreased alveolar formation (**a**,**b**), determined based on increased MLI (**d**) and decreased TVD (**e**) values. Treatment with recombinant furin improved alveolar development in hyperoxia-exposed mouse lungs (**c**–**e**, control, n = 7; hyperoxia, n = 6; furin treatment, n = 6, * *p* < 0.05). Scale bar, 50 μm. MLI, mean linear intercept; TVD, tissue volume density.

As shown in Figure 2, exposure to 85% oxygen for 10 d significantly reduced alveoli formation compared to the control (Figure 6a,b). Intraperitoneal recombinant furin administration at 2-d intervals starting at P2 in mice exposed to 85% oxygen rescued alveolar formation (Figure 6c), as confirmed via Lm and TVD measurements (Figure 6d,e, control, n = 7; 85% O_2_, n = 6; 85% O_2_ + furin, n = 6; *p* < 0.05).

## 4. Discussion

In this study, we showed that furin regulates alveolar formation through activating IGF-1R and its signaling. Prolonged exposure to hyperoxia reduced the expression of furin and its target protein, IGF-1R, resulting in decreased alveolar formation, mimicking the pathological features of BPD. Further, the administration of a furin inhibitor resulted in pathological changes in the lungs similar to those observed in the hyperoxia-induced BPD model, as indicated by the increased Lm and decreased TVD values. Furin inhibitor administration was also accompanied by the upregulation of pro-IGF-1R expression and reduced IGF-1R phosphorylation, suggesting that the inhibition of IGF-1R signaling is involved in BPD development. Finally, we also showed that recombinant furin administration to model animals partially alleviated the severity of lung injury. These results demonstrate that alveoli formation can be modified through modulating furin expression.

The establishment of surfactant replacement therapy and lung-protective strategies has resulted in improved respiratory outcomes in extremely preterm infants [1]. Consequently, instead of destructive lung injury, the arrest of alveolar septum formation has been newly recognized as a mainstay of the pathogenesis of “new BPD” [8]. Furin is reported to play an important role in the cleavage of precursor proteins, which are involved in the formation of secondary alveolar septa [15,16,17]. However, to date, associations between BPD and furin have not been investigated. We previously demonstrated an association between the phenotypic changes of myofibroblasts in the developing lung and furin-regulated PKGI activity [9]. Taken together, and considering the recent proposal regarding BPD pathogenesis [8], we hypothesized that furin activity is strongly associated with BPD development. 

Furin plays crucial roles in health and disease, as evidenced by its broad tissue distribution and insights gained from knockout mouse studies. These findings clearly indicate the indispensable role of furin in embryogenesis and developmental processes. While the involvement of furin in lung pathogenesis has been previously reported in conditions such as neoplasms, infections, and more recently in cystic fibrosis [22], its association with lung growth has not been demonstrated. To the best of our knowledge, this is the first study to demonstrate that furin expression is downregulated in the lungs of an in vivo hyperoxia-induced lung injury mouse model. Notably, our findings revealed that furin-positive cells are involved in septum formation, co-express SMA, a myofibroblast marker, and are decreased in chronic hyperoxia. Consistent with the recent proposed mechanisms of alveolarization [8], hyperoxia may inhibit myofibroblast-guided alveolar septum formation. Future studies on other BPD triggers, especially on myofibroblast dynamics in alveolar formation, are required to confirm our findings. 

Intraperitoneal furin inhibitor administration resulted in decreased alveolar formation, and this was associated with the suppression of IGF-1R activation, as well as the subsequent attenuation of its signaling. These results support the findings of previous studies, which showed that suppressing the activation of target proteins through furin inhibition in vascular smooth muscle cells ameliorates arteriosclerosis and vascular development in lung diseases [23,24].

Finally, we showed that intraperitoneal recombinant furin administration ameliorated the disturbance of alveolar formation in an animal model. Further, our results showed that hyperoxia-induced derangement of alveolarization was only partially alleviated through furin supplementation, suggesting that the timing and duration of furin deficiency may affect alveolarization. Intraperitoneal administration may affect organs other than the lungs given that furin is constitutively expressed throughout the body, and is essential for growth and differentiation [10]. To minimize the adverse effects of systemic furin administration, the safety and efficacy of trans-airway furin administration should be considered in future translational studies. Trans-tracheal drug administration is a common practice in neonatal care, as exemplified through the administration of pulmonary surfactants [25]. Further, the administration of drugs that modulate the activity of furin expressed in the lungs may be achieved via combination with an artificial pulmonary surfactant. For example, corticosteroid administration is more efficient than nebulization when combined with pulmonary surfactant [26]. 

This study had several limitations. Although we confirmed that reduced furin activity deteriorates alveolarization in developing mouse lungs, only wild-type mice were studied because furin-knockout mice are embryonically lethal [27]. This is due to hemodynamic insufficiency and defects in cardiac ventral closure. Therefore, future studies may consider using conditional knockout mice to modulate furin activity after birth. Second, even though the animal model used in this study is accepted as a standard BPD model [28], it does not fully mimic the clinical features of BPD. However, presenting these results in the model that has been employed in many studies facilitates comparison with previous works. Third, given that most of the proteins involved in alveolar formation are secreted proteins, the membrane protein, IGF-1R was selected as the target protein in this study. Considering that various factors contribute to the formation of alveolar septa, it will be necessary to examine whether the same pathological condition can be reproduced by modulating factors other than IGF-1R, whose activation is controlled via furin. Fourth, we employed a hybrid approach utilizing a hyperoxia-induced lung injury model in conjunction with primary cell culture to elucidate the mechanisms by which furin modulates IGF-IR. Notably, the immunoreactivity of pro IGF-IR appeared relatively faint in immunoblotting when utilizing whole lung lysate. In order to address this technical challenge, we utilized a myofibroblast primary culture to effectively demonstrate changes in the proprotein of IGF-IR. Further refinement may be necessary to address the difficulty of detecting relatively faint immunoreactivity on a blot when using whole lung samples. Finally, in this study, we used primary cultures of animal myofibroblasts but not those of human samples. Thus, the development of bridging biomarkers for both animal pups and human preterm infants may accelerate investigations regarding the mechanism of BPD development.

## 5. Conclusions

Using a mouse model of BPD and primary cultured cells, we showed that furin regulates alveolarization in immature lungs. The downregulation of the expression of furin and its target protein in the model resulted in decreased alveolarization. However, this condition was reversed via intraperitoneal furin administrations. This study provides a novel potential framework for understanding BPD pathogenesis. However, further analysis using knockout mice or other methods will be necessary to determine its overall effect on alveolar formation. With further investigations, furin administration to the lungs might be a novel therapeutic option for BPD in the future. Additionally, given the ubiquitous roles of furin in systemic organs other than the lung, the development of a strategy for its specific delivery to the lung, e.g., via transtracheal administration, should be considered. 

## Data Availability

All data generated or analyzed during this study are included in this article and Supplementary Materials. Further inquiries can be directed to the corresponding authors.

## Supplementary Materials

The following supporting information can be downloaded at: https://www.mdpi.com/article/10.3390/biom13111656/s1, Figure S1: Western blot images; Figure S2: Body weight changes in each animal experiment.

## References

[1] Stoll B.J., Hansen N.I., Bell E.F., Walsh M.C., Carlo W.A., Shankaran S., Laptook A.R., Sánchez P.J., Van Meurs K.P., Wyckoff M. (2015). Trends in Care Practices, Morbidity, and Mortality of Extremely Preterm Neonates, 1993–2012. JAMA.

[2] Jensen E.A., Edwards E.M., Greenberg L.T., Soll R.F., Ehret D.E.Y., Horbar J.D. (2021). Severity of Bronchopulmonary Dysplasia Among Very Preterm Infants in the United States. Pediatrics.

[3] Murthy K., Savani R.C., Lagatta J.M., Zaniletti I., Wadhawan R., Truog W., Grover T.R., Zhang H., Asselin J.M., Durand D.J. (2014). Predicting death or tracheostomy placement in infants with severe bronchopulmonary dysplasia. J. Perinatol..

[4] Isayama T., Lee S.K., Yang J., Lee D., Daspal S., Dunn M., Shah P.S., Canadian Neonatal Network, Canadian Neonatal Follow-Up Network Investigators (2017). Revisiting the Definition of Bronchopulmonary Dysplasia: Effect of Changing Panoply of Respiratory Support for Preterm Neonates. JAMA Pediatr..

[5] Schlapbach L.J., Adams M., Proietti E., Aebischer M., Grunt S., Borradori-Tolsa C., Bickle-Graz M., Bucher H.U., Latal B., Natalucci G. (2012). Outcome at two years of age in a Swiss national cohort of extremely preterm infants born between 2000 and 2008. BMC Pediatr..

[6] Michael Z., Spyropoulos F., Ghanta S., Christou H. (2018). Bronchopulmonary Dysplasia: An Update of Current Pharmacologic Therapies and New Approaches. Clin. Med. Insights Pediatr..

[7] Jobe A.H., Bancalari E. (2001). Bronchopulmonary dysplasia. Am. J. Respir. Crit. Care Med..

[8] Lignelli E., Palumbo F., Myti D., Morty R.E. (2019). Recent advances in our understanding of the mechanisms of lung alveolarization and bronchopulmonary dysplasia. Am. J. Physiol. Lung Cell Mol. Physiol..

[9] Kato S., Zhang R., Roberts J.D. (2013). Proprotein convertases play an important role in regulating PKGI endoproteolytic cleavage and nuclear transport. Am. J. Physiol. Lung Cell Mol. Physiol..

[10] Seidah N.G., Prat A. (2012). The biology and therapeutic targeting of the proprotein convertases. Nat. Rev. Drug Discov..

[11] Bassi D.E., Lopez De Cicco R., Mahloogi H., Zucker S., Thomas G., Klein-Szanto A.J. (2001). Furin inhibition results in absent or decreased invasiveness and tumorigenicity of human cancer cells. Proc. Natl. Acad. Sci. USA.

[12] Blanchette F., Day R., Dong W., Laprise M.H., Dubois C.M. (1997). TGFbeta1 regulates gene expression of its own converting enzyme furin. J. Clin. Investig..

[13] Hallenberger S., Bosch V., Angliker H., Shaw E., Klenk H.D., Garten W. (1992). Inhibition of furin-mediated cleavage activation of HIV-1 glycoprotein gp160. Nature.

[14] Stawowy P., Fleck E. (2005). Proprotein convertases furin and PC5: Targeting atherosclerosis and restenosis at multiple levels. J. Mol. Med..

[15] Dubois C.M., Laprise M.H., Blanchette F., Gentry L.E., Leduc R. (1995). Processing of transforming growth factor beta 1 precursor by human furin convertase. J. Biol. Chem..

[16] Duguay S.J. (1999). Post-translational processing of insulin-like growth factors. Horm. Metab. Res..

[17] Siegfried G., Khatib A.M., Benjannet S., Chrétien M., Seidah N.G. (2003). The proteolytic processing of pro-platelet-derived growth factor-A at RRKR(86) by members of the proprotein convertase family is functionally correlated to platelet-derived growth factor-A-induced functions and tumorigenicity. Cancer Res..

[18] Kawai S., Takagi Y., Kaneko S., Kurosawa T. (2011). Effect of three types of mixed anesthetic agents alternate to ketamine in mice. Exp. Anim..

[19] Hsia C.C., Hyde D.M., Ochs M., Weibel E.R. (2010). An official research policy statement of the American Thoracic Society/European Respiratory Society: Standards for quantitative assessment of lung structure. Am. J. Respir. Crit. Care Med..

[20] Suzuki T., Sato Y., Yamamoto H., Kato T., Kitase Y., Ueda K., Mimatsu H., Sugiyama Y., Onoda A., Saito S. (2020). Mesenchymal stem/stromal cells stably transduced with an inhibitor of CC chemokine ligand 2 ameliorate bronchopulmonary dysplasia and pulmonary hypertension. Cytotherapy.

[21] Bachiller P.R., Nakanishi H., Roberts J.D. (2010). Transforming growth factor-beta modulates the expression of nitric oxide signaling enzymes in the injured developing lung and in vascular smooth muscle cells. Am. J. Physiol. Lung Cell Mol. Physiol..

[22] Ornatowski W., Poschet J.F., Perkett E., Taylor-Cousar J.L., Deretic V. (2007). Elevated furin levels in human cystic fibrosis cells result in hypersusceptibility to exotoxin A–induced cytotoxicity. J. Clin. Investig..

[23] Kato S., Chen J., Cornog K.H., Zhang H., Roberts J.D. (2015). The Golgi apparatus regulates cGMP-dependent protein kinase I compartmentation and proteolysis. Am. J. Physiol. Cell Physiol..

[24] Stawowy P., Kallisch H., Kilimnik A., Margeta C., Seidah N.G., Chrétien M., Fleck E., Graf K. (2004). Proprotein convertases regulate insulin-like growth factor 1-induced membrane-type 1 matrix metalloproteinase in VSMCs via endoproteolytic activation of the insulin-like growth factor-1 receptor. Biochem. Biophys. Res. Commun..

[25] Yeh T.F., Chen C.M., Wu S.Y., Husan Z., Li T.C., Hsieh W.S., Tsai C.H., Lin H.C. (2016). Intratracheal Administration of Budesonide/Surfactant to Prevent Bronchopulmonary Dysplasia. Am. J. Respir. Crit. Care Med..

[26] Bassler D. (2015). Inhalation or instillation of steroids for the prevention of bronchopulmonary dysplasia. Neonatology.

[27] Scamuffa N., Calvo F., Chrétien M., Seidah N.G., Khatib A.M. (2006). Proprotein convertases: Lessons from knockouts. FASEB J..

[28] Nardiello C., Mižíková I., Morty R.E. (2017). Looking ahead: Where to next for animal models of bronchopulmonary dysplasia?. Cell Tissue Res..

