# Supplementary material for: Furin Regulates the Alveolarization of Neonatal Lungs in a Mouse Model of Hyperoxic Lung Injury
Source: Biomolecules. 2023 Nov 16;13(11):1656. doi: 10.3390/biom13111656 (PMC10669361; doi:10.3390/biom13111656)
Supplement: Supplementary file 1 [file biomolecules-13-01656-s001.zip › biomolecules-2670807-supplementary.pdf]

Supplementary figure S1

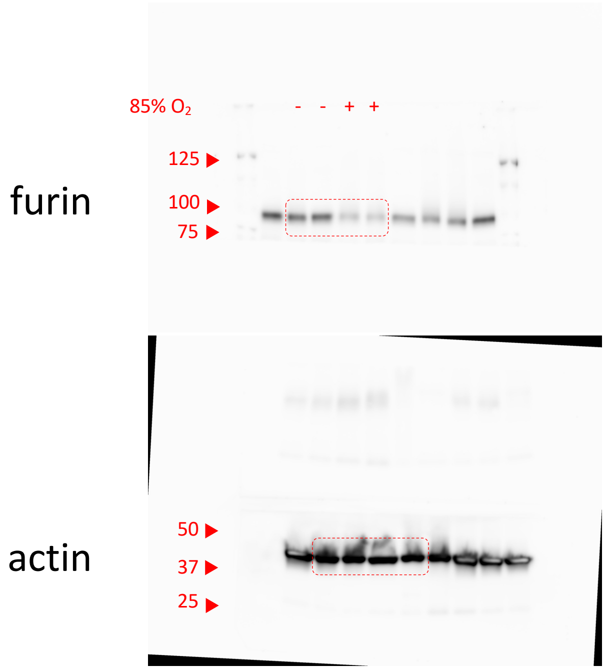

Original blots of Figure 2(e)

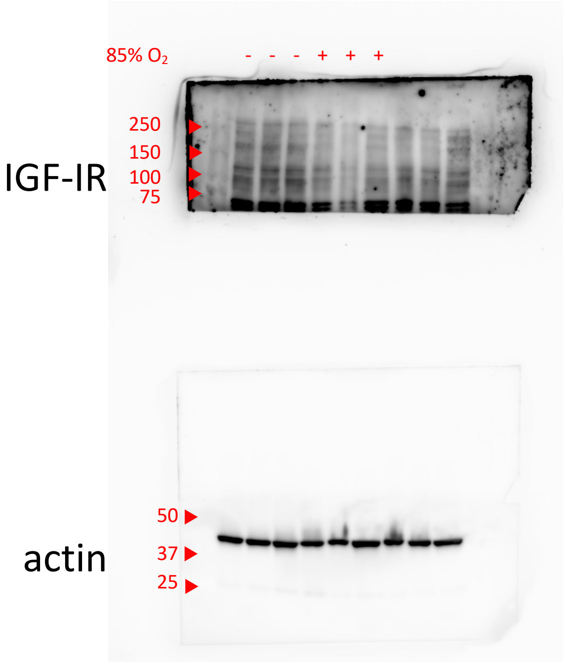

Original blots of Figure 3(a)

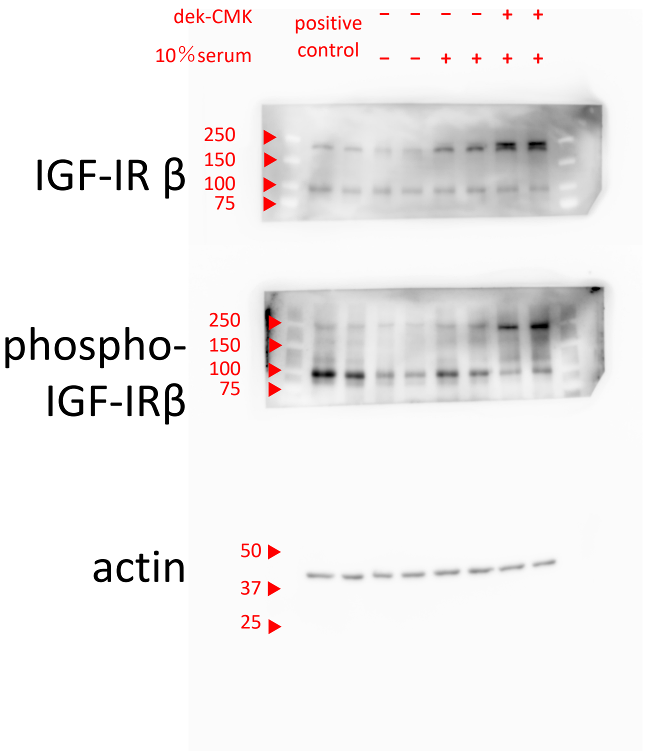

Original blots of Figure 5

Supplementary Figure S2

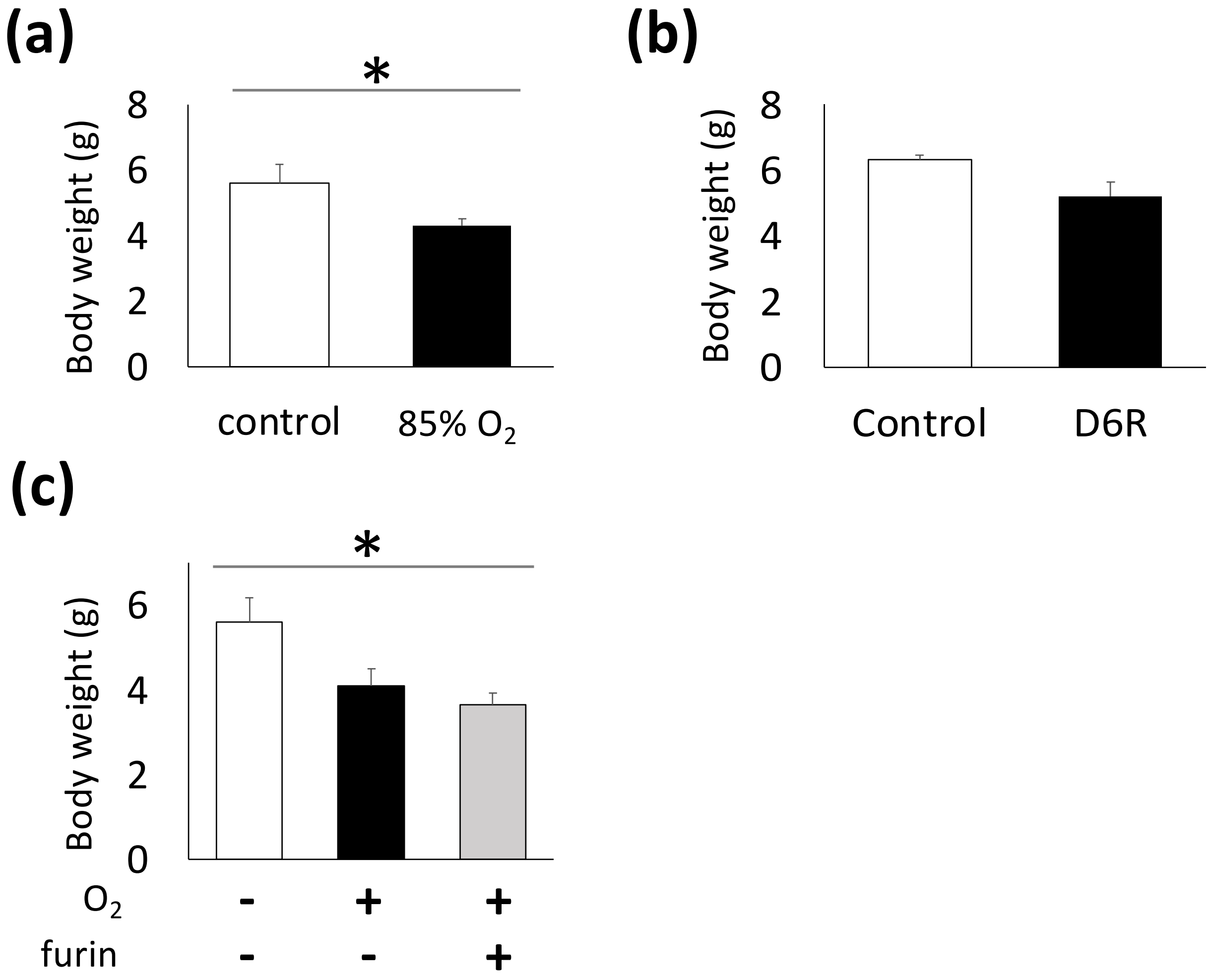

Figure S2. Mouse pups exposed to 85% O_2 for 10 days exhibited a significant decrease in body weight compared to the control mouse pups (a), (control, n=5; hyperoxia, n = 5; *$p < 0.05$). However, administration of Hexa-D Arginine showed lower body weight at P10, but failed to reach a significance (b), (control, n=5; D6R, n = 5) . Similarly, administration of recombinant furin to mice exposed to 85% O_2 did not show changes in body weight compared to the mice exposed to 85% O_2 treated with PBS (c), (control, n=5; hyperoxia, n = 5; hyperoxia + furin, n=5) .
